# Around the Hospitals

**Published:** 1893-02-11

**Authors:** 


### AROUND THE HOSPITALS.

**The Brighton Hospital for Women** was able to put forward some striking facts in its favour on the occasion of its sixty-second annual meeting. A great deal has been said about its work recently, and a special appeal has been made by the Mayor. The growth of the institution has been steady, and its claims to usefulness amply proved. Owing to careful management there is a small credit balance on the accounts, but the requirements of the institution need more funds than are necessary to cover current expenses. The rebuilding of the out-patients' department is indispensable if the hospital is to carry on its work effectually. If the sympathies of the many wealthy visitors to Brighton as well as those of the inhabitants be enlisted, we feel sure we shall hear shortly that the £800 necessary has been promptly subscribed.

**The Peterborough Infirmary** held its annual meeting recently. An important addition to the institution was announced as about to be made through the generosity of two ladies who desire to present a new wing as a memorial to their late brother. The new wing is to be devoted to the women's side of the infirmary, as improvement is most needed in that direction. It is to be hoped that the example of these generous donors will stimulate the inhabitants of Peterborough to fresh efforts for their hospital. The record of the past year shows a deficit in the accounts. This deficit would have been still greater had not, owing to careful management, a large decrease in the expenditure been also made during the year. Good management should be encouraged, and we doubt not that people in Peterborough will acknowledge this and heartily respond by cordial and increased support.

**The Manchester Southern Hospital** for the Diseases of Women and Children is the only one of its kind in the city, receiving as it does maternity in-patients. The usefulness of the institution is thoroughly established, and during the present year an increase in the accommodation has been made. Unfortunately a corresponding increase in the finances of the hospital has not shown itself, whilst improved quarters for the out-patients are very essential. The hospital forms an admirable school, both for students and midwives, and its advantages are increasingly in requisition, so much so that an enlargement of the students' department will soon be imperative. The hospital ministers, through the aid of competent midwives, to the needs of patients in their own homes, and here, too, the work is becoming so large that the ladies' committee state in their report that more supervision should now be provided. This is a very important point, and we have no doubt but that it will be recognized by the hospital authorities.

